# Topological non-Hermitian origin of surface Maxwell waves

**DOI:** 10.1038/s41467-019-08397-6

**Published:** 2019-02-04

**Authors:** Konstantin Y. Bliokh, Daniel Leykam, Max Lein, Franco Nori

**Affiliations:** 1 Theoretical Quantum Physics Laboratory, RIKEN Cluster for Pioneering Research, Wako-shi, Saitama 351-0198 Japan; 2 Nonlinear Physics Centre, RSPE, The Australian National University, Canberra, ACT 0200 Australia; 3 Center for Theoretical Physics of Complex Systems, Institute for Basic Science (IBS), Daejeon, 34126 Republic of Korea; 4 Advanced Institute of Materials Research, Tohoku University, Sendai, 980-8577 Japan; 5 Physics Department, University of Michigan, Ann Arbor, MI 48109-1040 USA

## Abstract

Maxwell electromagnetism, describing the wave properties of light, was formulated 150 years ago. More than 60 years ago it was shown that interfaces between optical media (including dielectrics, metals, negative-index materials) can support surface electromagnetic waves, which now play crucial roles in plasmonics, metamaterials, and nano-photonics. Here we show that surface Maxwell waves at interfaces between homogeneous isotropic media described by real permittivities and permeabilities have a topological origin explained by the bulk-boundary correspondence. Importantly, the topological classification is determined by the helicity operator, which is generically non-Hermitian even in lossless optical media. The corresponding topological invariant, which determines the number of surface modes, is a ${\Bbb Z}_4$ number (or a pair of ${\Bbb Z}_2$ numbers) describing the winding of the complex helicity spectrum across the interface. Our theory provides a new twist and insights for several areas of wave physics: Maxwell electromagnetism, topological quantum states, non-Hermitian wave physics, and metamaterials.

## Abstract (web summary)

Electromagnetic surface waves, derived from Maxwell theory, underpin many optical effects and applications. Here, Bliokh et al. show that surface waves at interfaces between isotropic media have a topological origin described by the non-Hermitian helicity operator and bulk-boundary correspondence.

## Introduction

Classical and quantum waves underlie the most fundamental entities in nature: light, sound, fields, and matter. Recently, an important role of topology in wave systems was revealed, describing the appearance of surface waves at interfaces between topologically different media^1–3^. This brought about the explanation of various physical phenomena (e.g., the quantum Hall effect^4,5^), the prediction of new phenomena (e.g., topological insulators^1,2^), and eventually resulted in the Nobel Prize in physics in 2016. While it was initially believed that topological effects are particular to quantum systems, they are universal wave phenomena, which since then have been realized in a wide range of classical waves, including electromagnetic^6–8^, acoustic^9^, mechanical^10^, and hydrodynamic^11^ systems.

Optics and electromagnetism provide one of the best platforms for studying fundamental relativistic wave phenomena, because classical Maxwell equations represent relativistic wave equations for massless spin-1 particles, i.e., photons within the first-quantization approach^12–14^. (This explains the mathematical similarities to the Dirac equation, even though Maxwell equations describe classical electromagnetic fields.) Moreover, studies of surface electromagnetic waves at interfaces between different media resulted in the rapid development of several areas of modern photonics, such as plasmonics^15,16^ and negative-index metamaterials^17–19^. Not surprisingly, the discovery of topological wave phenomena generated the rapidly developing field of topological photonics^20,21^.

Topological electromagnetic modes have been predicted and demonstrated in rather complicated nanostructured metamaterials, which mimic condensed-matter crystals with topologically nontrivial electron Hamiltonians. This approach requires considerable engineering efforts and suffers from inevitable losses, imperfections, etc. In contrast, in this paper, we reveal nontrivial topological properties for the most basic form of Maxwell equations involving only isotropic lossless homogeneous media characterized by the permittivity *ε* and permeability *μ*.

In this work, we show that all surface Maxwell waves appearing at interfaces between media with different signs of *ε* and *μ* are topological in nature. Here the term “topological” is justified in two ways. First, we describe the bulk-boundary correspondence, where the number of surface modes is determined by the contrast of a topological bulk invariant across the interface^1–3,20,21^. Importantly, this bulk invariant originates from the helicity operator of photons in a medium. This is the central difference of our work as compared with previously described topological systems based on the Hamiltonian operator. Furthermore, this helicity operator is generically non-Hermitian^22,23^ and has purely imaginary eigenvalues in “metallic” media with *εμ* < 0^24^. The topological bulk invariant is a ${\Bbb{Z}}_{4}$ number (or a pair of ${\Bbb{Z}}_{2}$ numbers), which describe the phase of the gapped helicity spectrum in a medium. The winding of this spectrum across the interface exactly corresponds to the number of surface electromagnetic modes, which are zero-helicity transverse-electric (TE) or transverse-magnetic (TM) polarized waves. Second, we connect the topology of the bulk system to the topology of the parameter (*ε*,*μ*) space; this is analogous to earlier works^3,25,26^ in the condensed-matter context. For Maxwell waves, the parameter space is split into four simply connected quadrants excluding the *ε* = 0 and *μ* = 0 lines, where the helicity is ill-defined. The helicity-based topological bulk invariant labels these quadrants of the parameter space. In addition to the topological invariant that provides the number of surface modes, we introduce a pair of non-topological ${\Bbb{Z}}_{2}$ indices, which separate the zones of the TE and TM polarizations in the phase diagram of surface modes.

Our non-Hermitian topological theory allows us to fully explain the nontrivial phase diagram of Maxwell surface modes, which includes well-known examples of surface plasmon-polaritons at metal-dielectric and negative-index interfaces, and to augment it with previously overlooked evanescent surface waves decaying along the propagation direction or/and in time. Although this diagram can be obtained from the standard Maxwell equations and boundary conditions, only the present topological theory explains why surface Maxwell modes of TE and TM polarizations exist in the corresponding regions of the parameter (*ε*,*μ*)-space.

## Results

### Winding of the helicity spectrum of photons in a medium

**Fig. 1:**
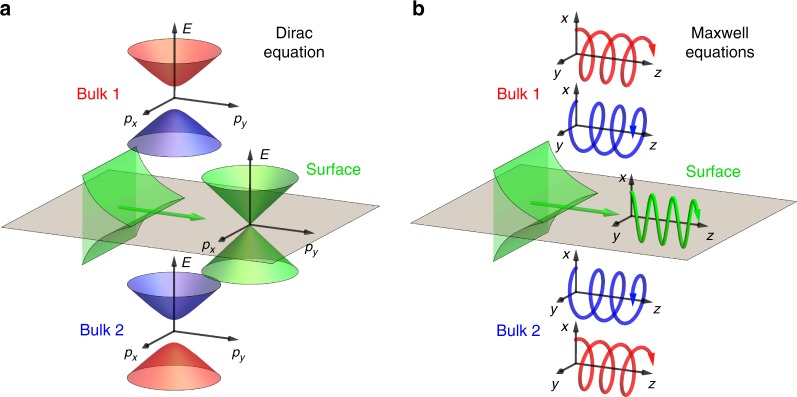
Schematics of topological surface modes in the Dirac and Maxwell equations. **a** The Dirac equation with a finite mass *m* is characterized by the gapped bulk spectrum *E*(**p**). An interface between “media” with opposite-sign masses ±*m*, and bulk spectra (schematically shown in red and blue), supports topological surface modes with massless spectrum (shown in green)^1–3,27^. **b** Maxwell equations possess massless bulk spectra (not shown here), which are double-degenerate with respect to opposite helicity states. These bulk helicity eigenmodes have opposite circular polarizations, i.e., chiral spatial distributions of the electric or magnetic field (shown in red and blue here). An interface between two media with different helicity properties (controlled by the signs of the permittivity *ε* and permeability *μ* of the medium) supports zero-helicity surface waves with transverse-electric or transverse-magnetic linear polarizations (shown in green)^15–19^

**Fig. 2:**
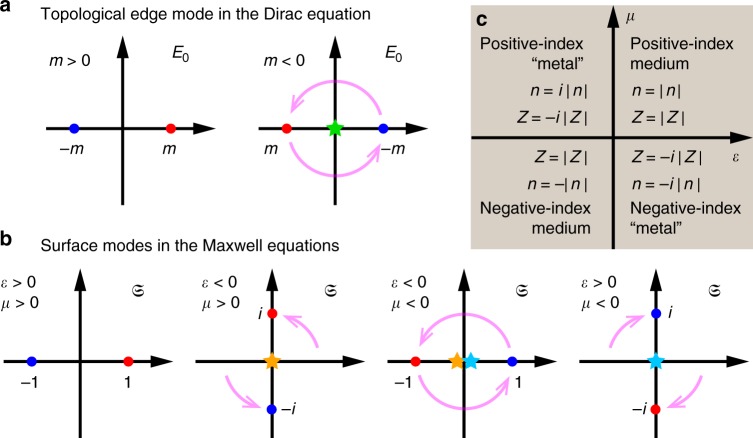
Winding of the energy and helicity spectra in the Dirac and Maxwell equations. **a** Changing the sign of the mass *m* in the Dirac equation is equivalent to a *π* rotation (shown by the arrows) of the rest-energy spectrum *E*_0_ ≡ *E*(**0**) = *m* in the complex-mass plane, which results in a single zero-mass surface mode (shown by the star symbol) protected by the topological ${\Bbb Z}_2$ winding number^1–3,27^. The dot and star symbols with their colors correspond to the rest-energy spectra of the bulk and surface modes shown in Fig. 1a. **b** Changing the signs of the permittivity *ε* and permeability *μ* in Maxwell equations produces ±*π*/2 and *π* rotations of the helicity spectrum in the complex helicity (${\frak S}$) plane Eq. (2). This results in the appearance of one or two zero-helicity (transverse-electric and transverse-magnetic) surface modes^15–19,34–36^ (shown by the star symbols) described by the topological ${\Bbb Z}_4$ number (3). **c** The medium-index diagram showing the signs of the refractive index *n* and impedance *Z* in four possible types of media (see Supplementary Note 1)

We start with the simplest example of topological surface modes, namely, the Jackiw-Rebbi edge states in the Dirac equation^1–3,27^. The bulk spectrum $E\left( {\mathbf{p}} \right) = \pm \sqrt {p^2 + m^2}$ of the Dirac equation is characterized by the energy gap 2*m* (we use *ħ* = *c* = 1 units) determined by the mass *m*. Then, an interface between two media with opposite masses *m*_1_ = −*m*_2_   supports a topological surface state with massless spectrum *E*^surf^ = ±*p*^surf^ (Fig. 1a). This edge mode is protected by the difference of the ${\Bbb{Z}}_{2}$ topological winding number $w = \frac{1}{2}{\mathop{\rm{sgn}}} \left( m \right)$ in the two media^1–3,27^. The transition between the two media can be viewed as a *π* rotation (i.e., winding) of the rest energies *E*_0_ ≡ *E*(**0**) = ±*m* → ±*e*^*iπ*^*m* = ∓*m* in the complex-energy (mass) plane (Fig. 2a), which illuminates the Möbius-strip-like ${\Bbb{Z}}_{2}$ topology.

Consider now electromagnetic waves (photons) described by the source-free Maxwell equations. Photons do not have mass but they possess another fundamental property: helicity, which can be associated with the projection of the photon’s spin **S** onto the direction of its momentum: ${\frak S} = {\mathbf{S}} \cdot {\mathbf{p}}{\mathrm{/}}\left| {\mathbf{p}} \right|$^24,28–31^. It is known that the helicity of free-space photons has two eigenvalues *σ* = ±1, corresponding to the right-hand and left-hand circularly polarized waves (Fig. 1b), whereas the independent zero-helicity state is forbidden because of the transversality of electromagnetic waves^28^. Thus, one can say that Maxwell bulk eigenmodes are characterized by the helicity gap (Fig. 2b).

In this paper, we deal with Maxwell waves in isotropic lossless media characterized by a real-valued permittivity *ε* and permeability *μ*. The possible dispersion of these parameters does not affect most of our considerations and is neglected hereafter. We will also use the refractive index *n* and dimensionless impedance *Z* of the medium, with $\left| n \right| = \sqrt {\left| {\varepsilon \mu } \right|} ,\left| Z \right| = \sqrt {\left| {\mu {\mathrm{/}}\varepsilon } \right|}$, and the signs defined as shown in Fig. 2c for four possible types of media^18^. The opposite refractive-index signs in the positive-index and negative-index materials reflect the fact that the complex-energy flux (Poynting vector) and momentum (wavevector) are parallel and anti-parallel in such media (see Supplementary Note 1)^17,18^. The gapless bulk energy spectrum of electromagnetic waves is determined by the dispersion relation: *ω*^2^ = *k*^2^/(*εμ*) (*ω* is the frequency, **k** is the wavevector), so that the bulk modes are propagating in transparent media with *εμ* > 0, and become purely evanescent, with imaginary wavevector or frequency, in “metallic media” with *εμ* < 0.

Maxwell equations for monochromatic light in a medium can be written in a quantum-like form as a Weyl-type equation^12–14,24,31,32^:

1 $$\left( {{\hat{\mathbf S}} \cdot {\hat{\mathbf p}}} \right)\psi = - \omega \hat \sigma ^{({\mathrm{m}})}\psi ,\,\,\,\,\hat \sigma ^{({\mathrm{m}})} = \left( {\begin{array}{*{20}{c}} 0 & { - i\mu } \\ {i\varepsilon } & 0 \end{array}} \right),\,\,\,\,\psi = \left( {\begin{array}{*{20}{c}} {\mathbf{E}} \\ {\mathbf{H}} \end{array}} \right).$$

Here *ψ* is the six-component “wavefunction”, $\hat {\bf{p}} = - i\nabla$ is the momentum operator, $\hat {\bf{S}}$ is the vector of 3 × 3 spin-1 matrices, acting on the Cartesian components of the fields as $\hat {\bf{S}} \cdot \hat {\bf{p}} = \nabla \times$, whereas the matrix $\hat \sigma ^{\left( {\mathrm{m}} \right)}$, describing the properties of the medium, acts on the “electric-magnetic” degrees of freedom, i.e., intermixes the **E** and **H** fields. The presence of a medium modifies the scalar product in this quantum-like approach, so that $\left\langle \psi \right|\left. \psi \right\rangle = \tilde \psi ^\dagger \cdot \psi$ with the adjoint “left” vector being $\tilde \psi = \left( {\varepsilon {\mathbf{E}},\mu {\mathbf{H}}} \right)^T \equiv \left( {{\mathbf{D}},{\mathbf{B}}} \right)^T$^24,33^. Using this formalism, it was recently shown that the helicity remains a fundamental physical property of electromagnetic waves in isotropic dispersive media^24^. Consider circularly polarized plane waves *ψ*^(*σ*)^ with the electric field ${\mathbf{E}}^{(\sigma )} = \left( {1,i\sigma ,0} \right)\exp \left( {i\,{\mathbf{k}} \cdot {\mathbf{r}} - i{\kern 1pt} \omega t} \right)$ (*σ* = ±1 determines the sign of the circular polarization), and the corresponding magnetic field **H**^(*σ*)^ = −*iσZ*^−1^**E**^(*σ*)^ (see Supplementary Note 1). These are eigenmodes of the helicity operator in the medium, $\hat {\frak S}$^24^, $\hat {\frak S}\psi ^{\left( \sigma \right)} = {\frak S}\psi ^{\left( \sigma \right)}$, with complex eigenvalues:

2 $$\hat {\frak S} = - \frac{{\hat \sigma ^{({\mathrm{m}})}}}{{\left| n \right|}} = \left( {\begin{array}{*{20}{c}} 0 & {i\eta Z} \\ { - i\eta Z^{ - 1}} & 0 \end{array}} \right),\,\,\,\,\,{\frak S} = \eta \sigma .$$

Here *η* = *n*/|*n*| indicates the phase of the refractive index, and we note that imaginary helicity makes physical sense because the canonical momentum (wavevector) becomes imaginary (while the spin remains real) in metallic media^24^. Remarkably, the helicity ${\frak S}$ always equals 1 in absolute value, but its phase essentially depends on the signs of *ε* and *μ*, i.e., is different in the four types of optical media mentioned above. At the dividing lines *ε* = 0 and *μ* = 0, separating different phases, the helicity is ill-defined (as well as the diverging energy eigenvalue *ω*).

Thus, the “helicity gap” is always present in optical media (apart from the singular *ε* = 0 and *μ* = 0 cases), whereas the media with different signs of (*ε*,*μ*) are related by *π*/2, *π*, and −*π*/2 rotations in the complex helicity plane, as shown in Fig. 2b. This suggests that electromagnetic media are split into four topologically different classes, described by the topological bulk invariant, which is a ${\Bbb{Z}}_{4}$ number or, equivalently, a pair of ${\Bbb{Z}}_{2}$ numbers:

3 $$\begin{array}{l}w\left( {\varepsilon ,\mu } \right) = \frac{2}{\pi }{{\mathrm{Arg}}}\left[ {\eta \left( {\varepsilon ,\mu } \right)} \right]\,{\mathrm{or}}\,\\ \left\{ {w^{{\mathrm{TM}}}\left( {\varepsilon ,\mu } \right),w^{{\mathrm{TE}}}\left( {\varepsilon ,\mu } \right)} \right\} = \frac{1}{2}\left\{ {1 - {\mathop{\rm{sgn}}} \left( \varepsilon \right),1 - {\mathop{\rm{sgn}}} \left( \mu \right)} \right\}.\end{array}$$

Here the ${\Bbb{Z}}_{4}$ number *w* takes on values 0, ±1, 2 in the four types of media shown in Fig. 2c, while the ${\Bbb Z}_2$ numbers *w*^TM,TE^ take on values 0, 1. We will refer to the invariant *w* as the helicity winding number, because the contrast of this invariant between two optical media describe the winding of the complex helicity spectrum across the interface.

**Fig. 3:**
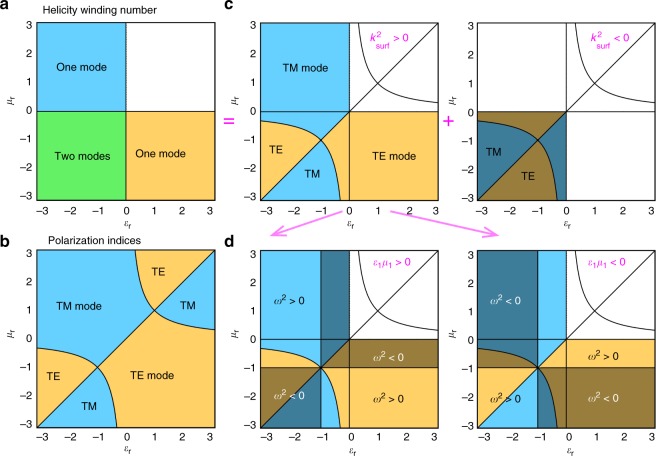
Phase diagrams of surface Maxwell waves. **a** Zones of the existence of zero, one, and two surface zero-helicity modes described by the topological helicity winding number Eq. (3) and the bulk-boundary correspondences Eqs. (4) and (5), see Fig. 2b. **b** Phase separation of the transverse-electric (TE) and transverse-magnetic (TM) modes described by the polarization indices Eq. (8). **c** The phase diagrams resulting from the combination of **a** and **b**. The two-mode quadrant (*ε*_r_ < 0,*μ*_r_ < 0) has both TE and TM modes in every point, but only one of these is propagating (i.e., having real wavevector $k_{{\mathrm{surf}}}$), while the other one is evanescent (having imaginary *k*_surf_). **d** Splitting the phase diagram (**c**) with real *k*_surf_ into zones with real (bright areas) and imaginary (dark areas) frequency $\omega _{{\mathrm{surf}}}$. These zones swap upon the inversion of the sign of the squared refractive index of the first medium, $n_1^2 = \varepsilon _1\mu _1$

Most importantly, interfaces between different media indeed support surface electromagnetic modes^15–19,34–36^, which are in agreement with the differences of the topological numbers, Eq. (3), across the interface. First, surface Maxwell modes always have zero helicity, ${\frak S}^{{\mathrm{surf}}} \equiv 0$, similarly to the zero-mass modes in topological insulators^1–3,27^ (Fig. 1). Indeed, surface Maxwell waves are either TE or TM, so that the product of the magnetic and electric wave fields, which determines the expectation value of the helicity operator Eq. (2), vanishes identically: ${\frak S} \propto {\mathbf{H}}^ \ast \cdot {\mathbf{E}} \equiv 0$^24,32^ (in agreement with this, the spin of these modes is orthogonal to the wavevector: **S** ⋅ **k** = 0^36^). Second, the number of TE and TM surface modes at the interface is exactly determined by the differences of the topological numbers Eq. (3):

4 $$N_{{\mathrm{surf}}} = \left| {w\left( {\varepsilon _2,\mu _2} \right) - w\left( {\varepsilon _1,\mu _1} \right)} \right| = \left| {w\left( {\varepsilon _{\mathrm{r}},\mu _{\mathrm{r}}} \right)} \right| = N_{{\mathrm{surf}}}^{{\mathrm{TE}}} + N_{{\mathrm{surf}}}^{{\mathrm{TM}}},$$

5 $$N_{{\mathrm{surf}}}^{{\mathrm{TM,TE}}} = \left| {w^{{\mathrm{TM,TE}}}{\kern 1pt} \left( {\varepsilon _2,\mu _2} \right) - w^{{\mathrm{TM,TE}}}\left( {\varepsilon _1,\mu _1} \right)} \right| = \left| {w^{{\mathrm{TM,TE}}}{\kern 1pt} \left( {\varepsilon _{{\mathrm{r}}},\mu _{\mathrm{r}}} \right)} \right|.$$

where the subscripts “1”, “2”, and “r” indicate the parameters of the two bulk media and the relative parameters characterizing the interface: (*ε*_r_,*μ*_r_) = (*ε*_2_/*ε*_1_,*μ*_2_/*μ*_1_). Note that in Eq. (4) the difference should be considered within the cyclic ${\Bbb Z}_4$ group: e.g. 2−(−1) = −1 rather than 3, because the helicity spectra of the corresponding media are related by a −*π*/2 rather than 3*π*/2 rotation. Equations (4) and (5) determine the bulk-boundary correspondences for the topological numbers Eq. (3) and surface Maxwell waves. In simple words, Eqs. (4) and (5) state that a single TM (TE) surface mode exists at an interface where only the permittivity *ε* (permeability *μ*) changes its sign, and two surface modes (TE and TM) exist at interfaces where both *ε* and *μ* change sign. This is shown in the phase diagram in Fig. 3a and is in perfect agreement with the properties of surface Maxwell waves known in plasmonics and metamaterials^15–19^.

Remarkably, the helicity winding number Eq. (3) can also be associated with the phase of the topological Chern number of photons^36^, which is also intimately related to the helicity and can become complex in metallic media that only support evanescent bulk modes. In free space (*ε* = *μ* = 1), the Berry curvature for photons is a monopole of charge *σ* at the origin of the momentum space: **F**^*σ*^ = *σ***k**/*k*^3^. Integrating it over momentum-space sphere yields the helicity-dependent Chern number *C*^*σ*^ = 2*σ*^36^. Extending this construction to isotropic media, we find that the momentum space becomes complex (assuming real frequency *ω*, the wavevectors **k** become imaginary in metallic media with *εμ* < 0). This results in the substitution **k**/*k* → *η***k**/*k*, and the Chern number becomes $C^\sigma = 2\eta {\kern 1pt} \sigma = 2{\frak S}$ (see Supplementary Note 2). Thus, transitions between media with different signs of *ε* or *μ* are accompanied by discrete changes of the phase of the complex Chern numbers, and the topological number Eq. (3) is determined by the phase of the spin Chern number: $w = \left( {2{\mathrm{/}}\pi } \right){\mathrm{Arg}}\left( {\sigma C^\sigma } \right)$. This illuminates the topological helicity properties of Maxwell equations in media and shows that these are quite different compared to Hermitian topological insulators with gapped energy spectra and real Chern numbers.

### Non-Hermitian features of the helicity and Maxwell equations

The above consideration reveals another fundamental peculiarity of the helicity-based description of photons in a medium. Namely, the helicity operator Eq. (2) is essentially non-Hermitian, as it is clearly seen from its purely imaginary spectrum in metallic media with *εμ* < 0. Therefore, the corresponding helicity-based form of Maxwell equations, Eq. (1), is also effectively non-Hermitian. Indeed, expanding the matrix $\hat \sigma ^{\mathrm{(m)}} = - \left| n \right|\hat {\frak S}$ in terms of the Hermitian Pauli matrices $\hat \sigma _i$, we write Maxwell equations as:

6 $$\left( {{\hat{\mathbf S}} \cdot {\hat{\mathbf p}}} \right)\psi = - \frac{\omega }{2}\left[ {\left( {\varepsilon + \mu } \right)\hat \sigma _2 + i\left( {\varepsilon - \mu } \right)\hat \sigma _1} \right]\psi .$$

Despite the non-Hermiticity of the operator in the right-hand side of this equation, its spectrum can be real (in transparent media with *εμ* > 0) because there is time-reversal symmetry $\hat K\hat \sigma _3$, where $\hat K$ is the complex conjugation^22,23,37^. Notably, it is known that Maxwell equations in a medium can be treated as a Hermitian energy eigenvalue problem, i.e., the frequency *ω* can always be chosen to be real (with the wavevector **k** becoming imaginary in metallic media)^6,24,33^. However, an important fact is missing in this Hermitian consideration with the modified inner product $\left\langle \psi \right|\left. \psi \right\rangle = \tilde \psi ^\dagger \cdot \psi$: it is valid for arbitrary (*ε*,*μ*) apart from the *ε* = 0 and *μ* = 0 values. The energy eigenvalues diverge at these values, *ω* → ∞, while the inner product coefficients vanish. Remarkably, these singular *ε* = 0 and *μ* = 0 values correspond to exceptional points^23,38^ of the operator $\hat \sigma ^{\left( {\mathrm{m}} \right)}$ in the helicity-based form (1) and (6) of Maxwell equations. The bulk helicity spectrum changes from real (*εμ* > 0) to imaginary (*εμ* < 0) at these points. Moreover, in each of the exceptional points, the bulk modes (*ψ* = (**E**,**H**)^*T*^∝(1,−*iσ*)^*T*^ are the eigenstates of $\hat \sigma _2$ in the vacuum) tend to a single “chiral” mode^38–40^ (the eigenstate of $\hat \sigma _3$): $\psi _{\mathop{\rm c}\nolimits} \propto \left( {1,0} \right)^T$ or *ψ*_c_ ∝ (0,1)^*T*^, having only an electric or magnetic field (see Supplementary Note 1). These chiral modes play crucial roles in the epsilon-near-zero or mu-near-zero materials^41^.

It is known in the theory of non-Hermitian systems that exceptional points are spectral degeneracies with nontrivial topological structure^23,38,42,43^. Namely, they have the topology of branch points, and it is impossible to introduce an unambiguous global labeling of eigenvalues in the vicinity of exceptional points. Thus, the parameter (*ε*,*μ*) space of Maxwell equations is actually split into four simply connected domains (quadrants) separated by the “exceptional lines” *ε* = 0 and *μ* = 0. The effective Hermitian description^6,24,33^ is possible in each of these domains but not globally over the whole (*ε*,*μ*) space. The helicity winding number (3) labels these topologically different quadrants of the parameter space, and has essentially non-Hermitian origin.

### Additional polarization indices

As mentioned above, the quantum-like formalism for Maxwell equations (1) determines the biorthogonal set of “right” and “left” eigenvectors *ψ* and $\tilde \psi$ ^24^. However, this choice is not unique. Alternatively, Maxwell equations can be formulated for the vectors *ψ*′ = (**E**,**B**)^*T*^ and $\tilde \psi \prime = \left( {{\bf{D}}{\mathrm{,}}{\bf{H}}} \right)^T$. In this case, Eq. (3) becomes:

7 $$\left( {{\hat{\mathbf S}} \cdot {\hat{\mathbf p}}} \right)\psi {\prime} = - \frac{\omega }{2}\left[ {\left( {\varepsilon \mu + 1} \right)\hat \sigma _2 + i\left( {\varepsilon \mu - 1} \right)\hat \sigma _1} \right]\psi \prime .$$

The non-Hermitian operator in the right-hand side of Eq. (7) has the same exceptional points as in Eq. (6). However, the Hermitian and non-Hermitian parts of the operators in Eqs. (6) and (7) differ from each other. Recently, analyzing topological edge modes in non-Hermitian quantum systems^44^, we showed that the sign of the non-Hermitian part of the operator can play an important role in this problem. For the operators in Eqs. (6) and (7), this results in a pair of ${\Bbb Z}_2$ indices:

8 $$v\left( {\varepsilon ,\mu } \right) = \frac{1}{2}\left\{ {{\mathop{\rm{sgn}}} (\varepsilon - \mu ),{\mathop{\rm{sgn}}} (\varepsilon \mu - 1)} \right\}.$$

As we show below, these indices describe the polarization TE/TM properties of surface modes, and therefore we will refer to these as “polarization indices”. Importantly, for a single medium, one can scale the electric and magnetic fields such that this will remove the non-Hermitian $\hat \sigma _1$-term in Eq. (6) or (7). In particular, scaling *ψ* = (*α***E**,*β***H**)^*T*^ with *β*/*α* = *Z* yields $- n\omega \hat \sigma _2\psi$ in the right-hand side of Eq. (6). However, such scaling is singular at the exceptional points *ε* = 0 and *μ* = 0, and, furthermore, it cannot remove the $\hat \sigma _1$-term simultaneously in two media. Applying the above scaling to the first medium, *β*/*α* = *Z*_1_, we find that the Maxwell equations in the second medium are given by Eqs. (6) and (7) with the substitution

9 $$\left( {\varepsilon _2,\mu _2} \right) \to \left( {\varepsilon _{\mathrm{r}},\mu _{\mathrm{r}}} \right),\,\omega \to n_1\omega .$$

Thus, the fundamental interface properties and surface modes must depend on the polarization indices Eq. (8) involving the relative permittivity and permeability: *v*(*ε*_r_,*μ*_r_). In contrast to the topological numbers Eq. (3) and the bulk-boundary Eqs. (4) and (5), the polarization indices of the relative interface parameters, *v*(*ε*_r_,*μ*_r_), cannot be expressed via differences of the corresponding bulk indices *v*(*ε*_1_,*μ*_1_) and *v*(*ε*_2_,*μ*_2_). This shows that the polarization indices Eq. (8) are not topological numbers, and there is no bulk-boundary correspondence for these. The role of these indices is revealed below.

### Phase diagrams for surface Maxwell waves

The detailed phase diagram of surface Maxwell modes can now be constructed using the topological invariants Eq. (3) with the bulk-boundary correspondences Eqs. (4) and (5), augmented by the polarization indices Eq. (8) and simple symmetry arguments. First, as it was mentioned above, the helicity winding number Eq. (3), *w*(*ε*_r_,*μ*_r_), yields a diagram in the (*ε*_r_,*μ*_*r*_)-plane (Fig. 3a), which determines the number of surface modes according to Eqs. (4) and (5). These modes must have vanishing helicity: ${\frak S}^{{\mathrm{surf}}} \propto {\mathbf{H}}^ \ast \cdot {\mathbf{E}} \equiv 0$. Taking into account the symmetry of a planar interface between two isotropic media implies that the surface modes must be TE or TM polarized, i.e., having electric and magnetic fields parallel and perpendicular to the interface and orthogonal to each other.

Second, the indices *v*(*ε*_r_,*μ*_r_) ≡ {*v*_1_,*v*_2_} determine the separation between the TE and TM phases. Indeed, from the dual symmetry between the electric and magnetic quantities (*ε*_r_ ↔ *μ*_r_, TE ↔ TM) and the spatial inversion symmetry, which exchanges the two media, 1 ↔ 2, and produces the substitution (*ε*_r_,*μ*_r_) → (1/*ε*_r_,1/*μ*_r_), one can conclude that the TE and TM modes must swap upon the sign flip of the non-Hermitian indices Eq. (8): *v*_1_ → −*v*_1_ or *v*_2_ → −*v*_2_. This results in the diagram (Fig. 3b), where the lines *ε*_r_ = *μ*_r_ and *ε*_r_*μ*_r_ = 1 divide the (*ε*_r_,*μ*_r_)-plane into alternating zones of TE and TM polarizations.

Note that according to the helicity winding diagram (Fig. 3a), both TE and TM surface waves exist at every point of the two-mode zone (*ε*_r_ < 0, *μ*_r_ < 0), but only one of these modes is shown in Fig. 3b. Showing both of these modes results in the two diagrams in Fig. 3c, but only the first diagram corresponds to the propagating surface modes. Indeed, direct calculations show that the wavevectors of the surface modes have the form $k_{{\mathrm{surf}}} \propto \sqrt {v_1v_2}$ and $k_{{\mathrm{surf}}} \propto \sqrt { - v_1v_2}$ for the TM and TE polarizations, respectively (see Supplementary Note 3). Hence one of these is always real (propagating modes in the first diagram Fig. 3(c)) while the other one is imaginary (evanescent surface modes in the second diagram (Fig. 3c)). Although these evanescent surface modes have never been considered before, these are observable, e.g., in the near-field scattering of surface electromagnetic waves. Furthermore, $k_{{\mathrm{surf}}} \to 0$ for both propagating and evanescent surface modes at interfaces involving epsilon-near-zero or mu-near-zero materials, where the contribution of evanescent surface modes can become crucial.

Finally, there is one more feature in the characterization of surface modes, which is not determined by the topological invariants Eq. (3) and polarizations indices Eq. (8). Up to now, we have allowed any frequencies $\omega _{{\mathrm{surf}}}$ of surface modes; however, because of the non-Hermitian character of the problem, these can also be either real or imaginary. In fact, the zones with real and imaginary frequencies are separated by the lines *ε*_r_ = −1 and *μ*_r_ = −1, which correspond to surface-plasmon resonances for a planar interface (see Supplementary Note 3). Furthermore, since we reduced the non-Hermitian interface problem (6) and (7) to the problem with relative parameters (*ε*_r_,*μ*_r_) and substitution *ω* → *n*_1_*ω*, as indicated in Eq. (9), the real-frequency and imaginary-frequency zones must swap upon the substitution $n_1^2 \to - n_1^2$. This splits the phase diagram of Fig. 3c into two diagrams for the $n_1^2 > 0$ and $n_1^2 < 0$ cases, as shown in Fig. 3d. Considering only propagating surface modes with real $\omega _{{\mathrm{surf}}}$ and $k_{{\mathrm{surf}}}$, we find that the phase diagrams of Fig. 3d exactly coincide with rather sophisticated diagrams previously obtained in refs. ^34–36^ by directly solving Maxwell equations. Importantly, the imaginary-*k* and imaginary-*ω* surface waves were ignored in the previous studies, which resulted in truncated phase diagrams. Taking these modes into account makes the picture complete and fully consistent with the simple diagram in Fig. 3a described by the topological helicity winding number Eq. (3) and bulk-boundary correspondence Eqs. (4) and (5).

## Discussion

We have shown that surface Maxwell waves have a fundamental topological origin, which is described by the helicity winding number (${\Bbb Z}_4$ or a pair of ${\Bbb Z}_2$ numbers) and bulk-boundary correspondence (Eqs. (3)–(5)). On the one hand, the underlying mechanism resembles the ${\Bbb Z}_2$ winding number for the Dirac topological insulators with opposite-mass interfaces^1–3^. On the other hand, the situation is fundamentally different because we deal with the winding of the helicity spectrum, ${\frak S}$, rather than that of the energy spectrum *E*. Moreover, the helicity operator in a medium is essentially non-Hermitian, and its spectrum can be either real or imaginary in lossless media. The helicity winding number labels the four topologically different quadrants of the parameter (*ε*,*μ*) space, which are separated by the exceptional points *ε* = 0 and *μ* = 0 of the helicity-like Maxwell operator in Eq. (6). In terms of momentum-space quantities, the non-Hermitian helicity leads to complex Chern numbers of photons in a medium, and their phase rather than the magnitude (as in the Hermitian case) corresponds to the helicity winding number.

The difference of the helicity winding number (3) between two media describes the number of surface Maxwell waves at the interface. Using different representations of the non-Hermitian helicity-based form of Maxwell equations, we introduce an additional pair of polarization ${\Bbb Z}_2$ indices (8). These are not topological numbers, they do not affect the number of surface modes, but these indices describe the separation of the TE and TM polarizations in the phase diagrams of surface modes (Fig. 3). Indeed, linking the polarization indices (8) to the phase diagram involves a spatial symmetry between the two media, which is broken when we replace the planar interface with a curved interface. In contrast, true topological phenomena are expected to survive at interfaces that break crystallographic symmetries.

Notably, due to their non-Hermitian origin, surface Maxwell waves are also essentially non-Hermitian modes. This means that these can have either real or imaginary frequencies and/or wave numbers. All previous studies of surface Maxwell waves considered only propagating surface waves with real parameters. This resulted in rather sophisticated and truncated phase diagrams^34–36^. Our theory augments this diagram with evanescent surface modes with imaginary parameters, which results in a very simple fundamental phase diagram (Fig. 3a) described solely by the topological helicity winding number (3).

Our theory provides new twists to several areas of wave physics: Maxwell electromagnetism, topological insulators, non-Hermitian quantum mechanics, and metamaterials. It shows that topological surface modes have been known and observed in electromagnetism long before the formulation of topological properties (e.g., surface plasmon-polaritons^15,16^). Furthermore, interfaces between the positive-index and negative-index metallic media (i.e., *ε* < 0, *μ* > 0 and *ε* > 0, *μ* < 0) provide “electromagnetic topological insulators” with no propagating bulk modes and topologically-protected surface modes^18,45^. We have also shown that macroscopic Maxwell equations in the helicity-based form naturally possess exceptional points in the (*ε*,*μ*)-space and “chiral” non-Hermitian bulk modes in these points, which correspond to the epsilon- and mu-near-zero materials^18,41^. Finally, we note that our approach can be applied to other wave equations, providing an efficient model for systems described by non-Hermitian massless wave equations with helical bulk modes.

## Supplementary information

Supplementary Information

## Data Availability

The data that support the findings of this study are available from the corresponding authors upon request.
