# Supplementary material for: Topological non-Hermitian origin of surface Maxwell waves
Source: Nat Commun. 2019 Feb 4;10:580. doi: 10.1038/s41467-019-08397-6 (PMC6362114; doi:10.1038/s41467-019-08397-6)
Supplement: Supplementary file 1 — Supplementary Information [file 41467_2019_8397_MOESM1_ESM.pdf]

Supplementary Information

Topological non-Hermitian origin of surface Maxwell waves

K. Y. Bliokh *et al*.

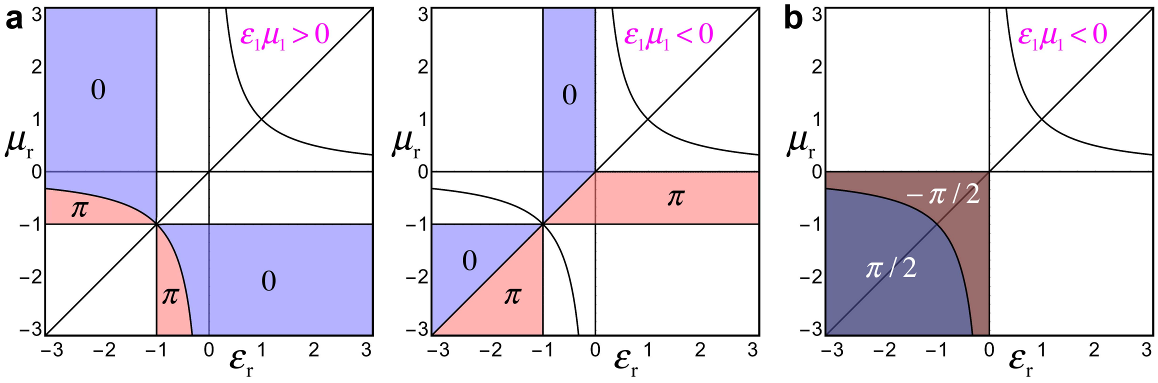

Supplementary Figure 1. Phases of the surface refractive index. Phases $\mathrm{Arg}(n_{\mathrm{surf}})$, Eq. (27), are shown for the (**a**) propagating ($k_z^2 > 0$) and (**b**) evanescent ($k_z^2 < 0$) surface modes with real frequencies, $\omega^2 > 0$. The blue (pink) zones correspond to the "right-handed" ("left-handed") surface modes with parallel (anti-parallel) wavevector and complex Poynting vector (26). All these diagrams are plotted for the $\mu_1 > 0$ case. Simultaneously flipping the signs of μ_1 and ε_1 swaps the "blue" and "pink" zones in these diagrams, i.e., changes the phase of n_{surf} by π.

Supplementary Note 1. Bulk modes of Maxwell equations

We consider monochromatic wave solutions of Maxwell's equations, which are described by complex electric and magnetic field amplitudes $\mathbf{E}(\mathbf{r})$ and $\mathbf{H}(\mathbf{r})$. The corresponding real fields are given by $\mathrm{Re}\left[\mathbf{E}(\mathbf{r})e^{-i\omega t}\right]$ and $\mathrm{Re}\left[\mathbf{H}(\mathbf{r})e^{-i\omega t}\right]$, where ω is the wave frequency. Maxwell's equations in an isotropic lossless homogeneous optical medium read:

$$\mu\omega\mathbf{H} = -i\nabla\times\mathbf{E}, \quad \varepsilon\omega\mathbf{E} = i\nabla\times\mathbf{H}, \quad \nabla\cdot\mathbf{H} = \nabla\cdot\mathbf{E} = 0, \tag{1}$$

where ε and μ are the real-valued permittivity and permeability of the medium.

Despite the medium being lossless, the first pair of these equations can be written in the non-Hermitian helicity-based form (1) or (6) of the main text:

$$\left(\hat{\mathbf{S}}\cdot\hat{\mathbf{p}}\right)\psi = -\frac{\omega}{2}\left[(\varepsilon+\mu)\hat{\sigma}_2 + i(\varepsilon-\mu)\hat{\sigma}_1\right]\psi\,. \tag{2}$$

Here, $\psi = (\mathbf{E},\mathbf{H})^T$ is the 6-component "wavefunction", and

$$\hat{\sigma}_1 = \begin{pmatrix} 0 & 1 \\ 1 & 0 \end{pmatrix}, \quad \hat{\sigma}_2 = \begin{pmatrix} 0 & -i \\ i & 0 \end{pmatrix}, \quad \hat{\sigma}_3 = \begin{pmatrix} 1 & 0 \\ 0 & -1 \end{pmatrix}$$

are the Pauli matrices exchanging the electric and magnetic fields, whereas

$$\hat{S}_x = \begin{pmatrix} 0 & 0 & 0 \\ 0 & 0 & -i \\ 0 & i & 0 \end{pmatrix}, \quad \hat{S}_y = \begin{pmatrix} 0 & 0 & i \\ 0 & 0 & 0 \\ -i & 0 & 0 \end{pmatrix}, \quad \hat{S}_z = \begin{pmatrix} 0 & -i & 0 \\ i & 0 & 0 \\ 0 & 0 & 0 \end{pmatrix},$$

are the spin-1 matrices acting on the Cartesian components of the fields as $-i\left(\hat{\mathbf{S}}\cdot\nabla\right) = \nabla\times$.

The bulk dispersion for Eqs. (1) and (2) is

$$\omega^2 = k^2/(\varepsilon\mu), \tag{3}$$

which describes a massless spectrum with a Dirac point in the origin of the momentum $\mathbf{k}$-space [1]. At the same time, the dispersion (3) shows that the signs of the permittivity and permeability determine different classes of optical media. For $\varepsilon\mu > 0$ the spectrum (3) is real and the medium is transparent, whereas for $\varepsilon\mu < 0$ the medium is opaque (we will refer to such medium as a "metal") and either the frequency or wavevector becomes *imaginary*. This exactly corresponds to the fact that the $\varepsilon = 0$ and $\mu = 0$ values determine the *exceptional points* [2,3] of the non-Hermitian operator in the right-hand side of Eq. (2). Below we show that the cases $(\varepsilon > 0, \mu > 0)$ and $(\varepsilon < 0, \mu < 0)$ or $(\varepsilon > 0, \mu < 0)$ and $(\varepsilon < 0, \mu > 0)$ are also topologically different.

Assuming propagation along the z-axis, the *circularly-polarized* bulk modes of Eqs. (1) can be written as:

$$\mathbf{E} \propto \frac{1}{\sqrt{2}}\begin{pmatrix} 1 \\ i\sigma \\ 0 \end{pmatrix}\exp\left(ik_z z\right), \quad \mathbf{H} = -i\sigma\frac{k_z}{\mu\omega}\mathbf{E}, \tag{4}$$

where $\sigma = \pm 1$ determines the sign of the polarization.

It is useful to introduce the complex *refractive index* n and the dimensionless *impedance* Z of the medium. These are determined by the relations

$$\varepsilon = \frac{n}{Z}, \qquad \mu = nZ, \tag{5}$$

which imply $n = \pm\sqrt{\varepsilon\mu}$ and $Z = \pm\sqrt{\mu/\varepsilon}$, but do not fix the signs of n and Z. In transparent media, these signs are fixed by distinguishing the *"right-handed" (positive-index)* and *"left-handed" (negative-index)* media, where the canonical momentum (wavevector) and energy flux (Poynting vector) are *parallel* and *antiparallel* to each other, respectively [4–6]. Since we consider a non-Hermitian model including "metallic" media with complex wavevectors, it is instructive to also consider the *complex* Poynting vector [7].

Assuming the frequency ω to be real, the modes (3) have real wavevectors k_z in transparent media and imaginary k_z in metallic media. The complex Poynting vector for these modes is:

$$\Pi_z \propto \left(\mathbf{E}^* \times \mathbf{H}\right)_z \propto \frac{k_z}{\mu}. \tag{6}$$

Thus, $\mathrm{sgn}(\mu)$ distinguishes "right-handed" and "left-handed" media with parallel and antiparallel canonical momentum and energy flux. Choosing the "+" sign for the refractive index of "right-handed" media, $n = +\sqrt{\varepsilon\mu}$, and the "–" sign for the "left-handed" media, $n = -\sqrt{\varepsilon\mu}$, and using Eq. (4), we arrive at the definition of the refractive indices and impedances in different media, as shown in Fig. 2(c). Using this definition, and setting $k_z = n\omega$, the relation (4) between the magnetic and electric fields of the bulk modes becomes $\mathbf{H} = -i\sigma Z^{-1}\mathbf{E}$.

We now consider the *helicity* of the bulk modes. As shown in detail in Ref. [8] (see also [9–11]), it is determined by the biorthogonal non-Hermitian formalism for Maxwell equations, where the "right" and "left" (adjoint) wavefunctions are given by $\psi = (\mathbf{E}, \mathbf{H})^T$ and $\tilde{\psi} = (\varepsilon\mathbf{E}, \mu\mathbf{H})^T \equiv (\mathbf{D}, \mathbf{B})^T$, whereas the helicity operator is:

$$\hat{\mathfrak{S}} = \begin{pmatrix} 0 & i\eta Z \\ -i\eta Z^{-1} & 0 \end{pmatrix}, \tag{7}$$

where $\eta = n/|n|$ is the parameter indicating the *phase* of the refractive index. Note that in the alternative non-Hermitian formalism of Eq. (7) in the main text, using the "right" and "left" wavefunctions $\psi' = (\mathbf{E}, \mathbf{B})^T$ and $\tilde{\psi} = (\mathbf{D}, \mathbf{H})^T$, the helicity operator becomes:

$$\hat{\mathfrak{S}}' = \begin{pmatrix} 0 & i\eta n^{-1} \\ -i\eta n & 0 \end{pmatrix}. \tag{8}$$

It is worth noticing that the conditions for Eqs. (7) and (8) to be Hermitian (i.e., proportional to the Pauli matrix $\hat{\sigma}_2$), $\eta^2 Z^2 = 1$ and $n^2 = 1$, determine the transitions in the pair of polarization indices ν, Eq. (8) in the main text. In any of these representations, the circularly-polarized plane waves (4)

with $\mathbf{H} = -i\sigma Z^{-1}\mathbf{E}$ are eigenmodes of the helicity operator with the eigenvalues Eq. (2) in the main text, which could also be written as expectation values:

$$\mathfrak{S} = \frac{\tilde{\psi}^* \hat{\mathfrak{S}} \psi}{\tilde{\psi}^* \psi} = \eta\sigma . \tag{9}$$

Note that the norm $\tilde{\psi}^*\psi = \left(\varepsilon + \mu |Z|^{-2}\right)|\mathbf{E}|^2$ vanishes in metallic media with $\varepsilon\mu < 0$. However, the ratio (9) remains finite. Furthermore, the inevitable dispersion in metals modifies this norm to $\left(\tilde{\varepsilon} + \tilde{\mu}|Z|^{-2}\right)|\mathbf{E}|^2$, where $(\tilde{\varepsilon},\tilde{\mu}) = (\varepsilon,\mu) + \omega\, d(\varepsilon,\mu)/d\omega$; this makes the norm positive-defined and does not affect the expectation value (9) [8].

Thus, the helicity (9) always equals to 1 in absolute value, while its phase, with respect to the vacuum value σ, is determined by the phase of the complex refractive index. This yields the topological classification of optical media by the "helicity $\mathbb{Z}_4$ winding number" $w = (2/\pi)\mathrm{Arg}(\eta)$, Eq. (3) in the main text, as discussed in the main text and shown in Fig. 2(b).

It is worth remarking on the unusual behavior of the bulk modes near the exceptional points $\varepsilon = 0$ and $\mu = 0$. In vacuum ($\varepsilon = \mu = 1$), the bulk modes with well-defined helicity are the two orthogonal eigenvectors of the $\hat{\sigma}_2$ Pauli matrix: $\psi = (\mathbf{E},\mathbf{H})^T \propto (1,\mp i)^T$. In the $\varepsilon = 0$ exceptional point, the impedance becomes infinite, and the two bulk modes coalesce to a *single "chiral" mode* [12–15] with only-magnetic field: $\psi_{\mathrm{c}} = (\mathbf{E},\mathbf{H})^T \propto (0,1)^T$. Here the magnetic field can still have two polarizations, and the "chirality" is assumed in the space of Pauli matrices $\hat{\sigma}_i$, i.e., the "chiral" mode is a single eigenvector of $\hat{\sigma}_3$. Such anomalous modes manifest themselves in *"epsilon-near-zero" materials* [16–18]. In the $\mu = 0$ exceptional point, the "chiral" mode has only the electric field: $\psi_{\mathrm{c}} = (\mathbf{E},\mathbf{H})^T \propto (1,0)^T$.

We also note that the non-Hermitian $\hat{\sigma}_1$-term in Eq. (2) can be removed by rescaling the electric and magnetic fields. Indeed, choosing $\psi = (\alpha\mathbf{E},\beta\mathbf{H})^T$ with $\beta/\alpha = Z$, the equation (2) becomes:

$$\left(\hat{\mathbf{S}}\cdot\hat{\mathbf{p}}\right)\psi = -n\omega\hat{\sigma}_2\psi . \tag{10}$$

However, this scaling is: (i) singular in the exceptional points, and (ii) cannot remove the non-Hermitian terms simultaneously in *two* media. In the case of two media, performing such scaling in the medium 1, results in the non-Hermitian equation for the medium 2:

$$\left(\hat{\mathbf{S}}\cdot\hat{\mathbf{p}}\right)\psi = -\frac{n_1\omega}{2}\left[\left(\varepsilon_{\mathrm{r}} + \mu_{\mathrm{r}}\right)\hat{\sigma}_2 + i\left(\varepsilon_{\mathrm{r}} - \mu_{\mathrm{r}}\right)\hat{\sigma}_1\right]\psi , \tag{11}$$

where n_1 is the refractive index of the first medium, whereas $\varepsilon_{\mathrm{r}} = \varepsilon_2/\varepsilon_1$ and $\mu_{\mathrm{r}} = \mu_2/\mu_1$ are the relative permittivity and permeability. Thus, the Maxwell interface can be considered as an interface between the "Hermitian vacuum", with the substitution $\omega \to n_1\omega$, and a "non-Hermitian medium" determined by the relative parameters ε_{r} and μ_{r}, Eqs. (9) in the main text.

Supplementary Note 2. Complex Chern numbers for photons in a medium

In most known topological wave systems, the topological numbers are associated with properties of the system *Hamiltonian* over momentum space. The helicity winding number, Eq. (3) in the main text, is a different topological construction, which is based on the behavior of the *helicity* operator over the parameter (ε,μ)-space. Nonetheless, it can also be related to more familiar *Chern numbers* defined via the integration of the bulk eigenmodes in momentum space.

The Chern numbers of *free-space* photons are well understood [1]. The free-space eigenmodes with well-defined energy and helicity are circularly-polarized plane waves propagating in an arbitrary direction determined by the wavevector $\mathbf{k}$. The transversality conditions $\mathbf{E}\cdot\mathbf{k}=\mathbf{H}\cdot\mathbf{k}=0$ make the polarization vectors effectively $\mathbf{k}$-dependent and generate the Berry connection and curvature in momentum space. The Berry connection has the form of a helicity-dependent monopole at the origin of momentum space [1,19–21]:

$$\mathbf{F}^{\sigma}=\sigma\frac{\mathbf{k}}{k^{3}}. \qquad (12)$$

Integrating this curvature over all directions, i.e., calculating the flux of the monopole field (12) through a sphere in momentum space, we obtain the helicity dependent Chern numbers of photons [1]:

$$C^{\sigma}=\frac{1}{2\pi}\oint\mathbf{F}^{\sigma}\cdot d^{2}\mathbf{k}=2\sigma. \qquad (13)$$

One can also introduce the *spin (helicity) Chern number* $C^{\rm spin}=\sum\limits_{\sigma=\pm1}\sigma C^{\sigma}=4$ [1,22,23].

Calculations in an isotropic lossless medium, using circularly-polarized plane-wave modes similar to (4), result in the same Berry curvature (12). This is because the Berry curvature (12) originates from properties of the circular-polarization vectors and transversality conditions, which remain unchanged in a homogeneous isotropic medium. Equation (12) in a medium can be formally derived using the wavefunction $\psi=(\mathbf{E},\mathbf{H})^{T}$, the "left wavefunction" $\tilde{\psi}=(\varepsilon\mathbf{E},\mu\mathbf{H})^{T}$, the Berry connection $\mathbf{A}^{\sigma}=-i\langle\tilde{\psi}|\nabla_{\mathbf{k}}|\psi\rangle/\langle\tilde{\psi}|\psi\rangle$, and curvature $\mathbf{F}^{\sigma}=\nabla_{\mathbf{k}}\times\mathbf{A}^{\sigma}$. The only modification of the Berry curvature as compared to the free-space case is the value of the wavevector $\mathbf{k}$, which changes between different media. In particular, in metallic media with $\varepsilon\mu<0$, the wavevectors become purely *imaginary*, assuming real frequency ω in Eq. (3). Thus, one can say that *the presence of a medium modifies the properties of momentum space but preserves the form of the Berry-curvature monopole (12)*.

The modification of the wavevector, compared with the free-space case, is described by the transformation $\mathbf{k}\to n\mathbf{k}$. To understand how this affects the topological properties of light, i.e., the Chern numbers (13), we note that the factor $1/k^{2}$ in the monopole (12) is compensated by the surface integration $d^{2}\mathbf{k}$ in Eq. (13). The remaining part of the monopole (12) is then transformed as

$$\sigma\frac{\mathbf{k}}{k}\to\sigma\frac{n}{|n|}\frac{\mathbf{k}}{k}=\eta\sigma\frac{\mathbf{k}}{k}=\mathfrak{S}\frac{\mathbf{k}}{k}. \qquad (14)$$

From here, it is easy to conclude that the Chern number (13) and spin (helicity) Chern number in a medium become

$$C^{\sigma} = 2\eta\sigma = 2\mathfrak{S}\,, \qquad C^{\text{spin}} = 4\eta\,. \tag{15}$$

Remarkably, the Chern numbers of photons in media become *complex*. This is caused by the *complexification of the momentum* $\mathbf{k}$*-space* (assuming the frequency ω to be real). Indeed, in free space, only the *direction* of the wavevector, $\mathbf{k}/k = \mathbf{k}/|\mathbf{k}| = \mathbf{k}/\sqrt{\mathbf{k}\cdot\mathbf{k}}$, matters. In a medium, both the direction and *phase* of the wavevector, $\text{Arg}\sqrt{\mathbf{k}\cdot\mathbf{k}}$, play roles. Therefore, in contrast to the majority of known topological wave systems, *the phase rather than magnitude of the Chern numbers* describe topological properties of Maxwell equations. While the magnitude is constant and unchanged between different media, the phase can vary and yields exactly the "*helicity winding number*", Eq. (3) in the main text:

$$w = \frac{2}{\pi}\text{Arg}\left(C^{\text{spin}}\right) = \frac{2}{\pi}\text{Arg}\left(\eta\right). \tag{16}$$

This describes the relation between the helicity winding number introduced in this work and the widely known Chern numbers. To the best of our knowledge, the system considered in this work, i.e., Maxwell equations in an isotropic lossless medium, provide the first example of a wave system with complex Chern numbers. Note that in the particular case of an interface between right-handed ($\varepsilon > 0$, $\mu > 0$, $\eta = 1$) and left-handed ($\varepsilon < 0$, $\mu < 0$, $\eta = -1$) transparent media, the presence of surface modes agrees with standard models involving real Chern numbers. Indeed, the spin Chern numbers in the two media equal $C^{\text{spin}} = 4$ and $C^{\text{spin}} = -4$, respectively. In the known quantum spin-Hall effect case for electrons [22,23], an interface with spin Chern numbers $C^{\text{spin}} = \pm 2$ results in a *single* edge state propagating in each direction. In the Maxwell case under consideration, the contrast of the spin Chern numbers is twice as large, and this results in *two* surface waves (TE and TM) propagating in a given direction at an interface between a right-handed and left-handed medium, see Fig. 3(a).

It is worthwhile to compare the above calculation to very recent studies [24,25] of edge states in non-Hermitian tight binding models, which appeared after the submission of this work. These papers identified non-Hermitian systems where the correct bulk-boundary correspondence does not involve bulk modes with purely real wavevectors $\mathbf{k}$ (i.e., propagating Bloch modes). Due to the "non-Hermitian skin effect", the bulk modes also become evanescent (acquiring complex wavevectors). In this case, Refs. [24,25] showed that *the number of edge states only changes at phase transitions where the bulk modes delocalize*. Our phase diagram Fig. 3(a) for the helicity winding number is fully consistent with these studies; in particular, $\mathbf{k}$ vanishes and the number of surface modes changes at the transition points $\varepsilon = 0$ and $\mu = 0$, implying a transition from localized to propagating bulk modes.

Supplementary Note 3. Surface modes of Maxwell equations

We now consider a planar $x = 0$ interface between two isotropic lossless homogeneous media:

$$\varepsilon, \mu = \begin{cases} \varepsilon_1, \mu_1 & \text{for } x > 0 \\ \varepsilon_2, \mu_2 & \text{for } x < 0 \end{cases} \tag{17}$$

We also use the relative permittivity and permeability $\varepsilon_{\rm r} = \varepsilon_2 / \varepsilon_1$ and $\mu_{\rm r} = \mu_2 / \mu_1$. Such an interface can support TE or TM surface waves [1,26–32]. Assuming the propagation along the z-axis, these waves are described by the 2D scalar wave equations:

$$\Delta E_y + \varepsilon\mu\omega^2 E_y = 0 \quad \text{(TE)},$$
$$\Delta H_y + \varepsilon\mu\omega^2 H_y = 0 \quad \text{(TM)}, \tag{18}$$

where $\Delta = \frac{\partial^2}{\partial x^2} + \frac{\partial^2}{\partial z^2}$ and the other nonzero field components are determined by

$$H_z = -\frac{i}{\mu\omega}\frac{\partial E_y}{\partial x}, \quad H_x = \frac{i}{\mu\omega}\frac{\partial E_y}{\partial z} \quad \text{(TE)},$$
$$E_z = \frac{i}{\varepsilon\omega}\frac{\partial H_y}{\partial x}, \quad E_x = -\frac{i}{\varepsilon\omega}\frac{\partial H_y}{\partial z} \quad \text{(TM)}. \tag{19}$$

We seek the modes localized at the interface, i.e., having the form

$$\left\{E_y, H_y\right\} \propto \exp\left(ik_z z - \kappa_{1,2}|x|\right), \quad \varepsilon_{1,2}\mu_{1,2}\omega^2 = k_z^2 - \kappa_{1,2}^2, \tag{20}$$

where $\kappa_{1,2} > 0$ are the real spatial-decay constants in the two media, E_y and H_y correspond to the TE and TM modes, subscripts "1" and "2" correspond to the fields in the two media, and we used the dispersion relation (3) in each of the media. Substituting Eqs. (20) into wave equations (18) and (19), and using the continuity of the tangential components $E_{y,z}$ and $H_{y,z}$ for the TE and TM modes, we obtain [1,30–32]:

$$\frac{\kappa_1}{\mu_1} + \frac{\kappa_2}{\mu_2} = 0 \quad \text{(TE)}, \qquad \frac{\kappa_1}{\varepsilon_1} + \frac{\kappa_2}{\varepsilon_2} = 0 \quad \text{(TM)}. \tag{21}$$

The surface Maxwell modes are fully described by the algebraic equations (20) and (21). First, since $\kappa_{1,2} > 0$ is the necessary condition of the surface-mode localization, it is evident from Eqs. (21) that: (i) there are no surface modes at an interface with $\left(\mu_{\rm r} > 0, \varepsilon_{\rm r} > 0\right)$; (ii) there can be both TE and TM modes at an interface with $\left(\mu_{\rm r} < 0, \varepsilon_{\rm r} < 0\right)$; (iii) there is only one TM mode at an interface with $\left(\mu_{\rm r} > 0, \varepsilon_{\rm r} < 0\right)$; and (iv) there is only one TE mode at an interface with $\left(\mu_{\rm r} < 0, \varepsilon_{\rm r} > 0\right)$. *This precisely coincides with the phase diagram in Fig. 3(a), which is described by the "helicity winding number"* $w\left(\varepsilon_{\rm r}, \mu_{\rm r}\right)$ *and bulk-boundary correspondence, Eqs. (3)–(5) in the*

main text. Moreover, it is easy to see that the expectation value of the helicity (7)–(9), determined by the product $\mathbf{H}^* \cdot \mathbf{E}$, vanishes identically for the TE and TM surface waves:

$$\mathfrak{S}_{\text{surf}} \equiv 0\,. \tag{22}$$

Second, solving equations (20) and (21), we obtain

$$k_z^2 = \kappa_1^2 \frac{\mu_{\text{r}}\left(\varepsilon_{\text{r}} - \mu_{\text{r}}\right)}{\varepsilon_{\text{r}}\mu_{\text{r}} - 1}, \quad \varepsilon_1\mu_1\omega^2 = \kappa_1^2 \frac{1-\mu_{\text{r}}^2}{\varepsilon_{\text{r}}\mu_{\text{r}} - 1} \quad \text{(TE)},$$

$$k_z^2 = \kappa_1^2 \frac{\varepsilon_{\text{r}}\left(\mu_{\text{r}} - \varepsilon_{\text{r}}\right)}{\varepsilon_{\text{r}}\mu_{\text{r}} - 1}, \quad \varepsilon_1\mu_1\omega^2 = \kappa_1^2 \frac{1-\varepsilon_{\text{r}}^2}{\varepsilon_{\text{r}}\mu_{\text{r}} - 1} \quad \text{(TM)}. \tag{23}$$

Note that the solutions (23) depend only on the relative parameters ε_{r} and μ_{r} with the substitution $\omega \to n_1\omega$, as indicated in Eq. (11). Most importantly, since $\mu_{\text{r}} < 0$ ($\varepsilon_{\text{r}} < 0$) for the TE (TM) modes, *the sign of k_z^2 is fully determined by the product of the two non-topological polarization indices, Eq. (8) in the main text, at the interface,* $v\left(\varepsilon_{\text{r}}, \mu_{\text{r}}\right) \equiv \left\{v_1, v_2\right\}$:

$$k_z^2 \propto -v_1 v_2 \quad \text{(TE)}, \qquad k_z^2 \propto v_1 v_2 \quad \text{(TM)}, \tag{24}$$

This shows that changing the sign of v_1 or v_2 corresponds to the swapping of the TE and TM modes. This is schematically shown in Fig. 3(b), where the $v_1 = 0$ and $v_2 = 0$ lines divide the $\left(\varepsilon_{\text{r}}, \mu_{\text{r}}\right)$-plane into alternating TE and TM zones. It should be remembered, however, that the "two-mode" zone with $\varepsilon_{\text{r}} < 0$ and $\mu_{\text{r}} < 0$ supports *both* TE and TM modes in every point, Fig. 3(a). Analyzing the expressions (24) with Eqs. (3) and (8) in the main text, we find that in the "one-mode" TE (TM) zone with $\varepsilon_{\text{r}} > 0$, $\mu_{\text{r}} < 0$ ($\varepsilon_{\text{r}} < 0$, $\mu_{\text{r}} > 0$), the non-Hermitian indices are $v_1 > 0$, $v_2 < 0$ ($v_1 > 0$, $v_2 < 0$), so that this mode is always *propagating*: $k_z^2 > 0$. At the same time, in the "two-mode" zone $\varepsilon_{\text{r}} < 0$ and $\mu_{\text{r}} < 0$, with simultaneously existing TE and TM modes, one of the modes is *propagating* ($k_z^2 > 0$), while the other one is *evanescent* ($k_z^2 < 0$). Crossing the line $v_1 = 0$ or $v_2 = 0$ in the "two-mode" zone switches the propagating TE (TM) mode to the evanescent TE (TM) mode and vice versa, as shown in Fig. 3(c).

Thus, the topological numbers, Eq. (3) in the main text, and polarization indices, Eq. (8) in the main text, fully control the domains of the existence and propagation properties, $\text{sgn}\left(k_z^2\right)$, of surface Maxwell waves. However, due to the non-Hermitian character of these modes, their frequencies ω can also be complex (either real or imaginary in lossless media). Equations (23) show that this behavior is *not* described by the above indices and involves additional frequency zeros in the $\varepsilon_{\text{r}} = -1$ and $\mu_{\text{r}} = -1$ points. These zeros divide the phase diagram of the propagating surface modes ($k_z^2 > 0$), Fig. 3(c), into real-frequency ($\omega^2 > 0$) and imaginary-frequency ($\omega^2 < 0$) zones, as shown in Fig. 3(d). Due to the $\varepsilon_1\mu_1$ prefactor in Eqs. (23), these zones are opposite for the transparent-first-medium and metallic-first-medium cases (this does not break the symmetry $1 \leftrightarrow 2$ between the two media because the coordinates $\left(\varepsilon_{\text{r}}, \mu_{\text{r}}\right)$ are asymmetric). Considering only

propagating surface waves with real frequencies, we obtain the phase diagrams previously found in Refs. [1,31,32].

For completeness, we also find the complex energy flux (Poynting vector) in surface Maxwell modes. Locally, it is given by

$$\Pi_z \propto -E_y^* H_x = \frac{k_z}{\mu\omega}\left|E_y\right|^2 \quad \text{(TE)}, \qquad \Pi_z \propto E_x^* H_y = \frac{k_z^*}{\varepsilon\omega}\left|H_y\right|^2 \quad \text{(TM)}. \tag{25}$$

Since its direction is opposite in the two media, it makes sense to calculate the integral Poynting vector for the localized surface wave. Integrating Eqs. (25) across the interface, $\langle ... \rangle = \int ...dx$, we find

$$\langle \Pi_z \rangle \propto \frac{k_z}{\mu_1 \omega \kappa_1}\left(1-\mu_{\rm r}^{-2}\right)\left|E_y\right|^2 \quad \text{(TE)}, \qquad \langle \Pi_z \rangle \propto \frac{k_z^*}{\varepsilon_1 \omega \kappa_1}\left(1-\varepsilon_{\rm r}^{-2}\right)\left|H_y\right|^2 \quad \text{(TM)}. \tag{26}$$

Similar to the bulk modes, the Poynting vector (26) is either parallel or anti-parallel to the wavevector k_z, which distinguishes the "right-handed" and "left-handed" surface waves [31,32]. By analogy with the bulk modes, one can also define the "surface refractive index", $n_{\rm surf}^2 = k_z^2 / \omega^2$, using the dispersion relations (23). This yields

$$n_{\rm surf}^2 = \varepsilon_1 \mu_1 \frac{\mu_{\rm r}\left(\varepsilon_{\rm r} - \mu_{\rm r}\right)}{1-\mu_{\rm r}^2} \quad \text{(TE)}, \qquad n_{\rm surf}^2 = \varepsilon_1 \mu_1 \frac{\varepsilon_{\rm r}\left(\mu_{\rm r} - \varepsilon_{\rm r}\right)}{1-\varepsilon_{\rm r}^2} \quad \text{(TM)}, \tag{27}$$

One can fix the signs (phases) of these indices, using the mutual direction of the complex Poynting vector (26) and the wavevector k_z. However, it should be noticed that the time-averaged Poynting vector (25) and (26) is well-defined only for surface waves with *real frequencies* ω.

Supplementary Figure 1 shows the phase diagrams for the surface refractive index (27) for the propagating ($k_z^2 > 0$) and evanescent ($k_z^2 < 0$) surface modes. Note that our diagrams separating the "right-handed" and "left-handed" surface modes coincide with those in Ref. [32] in the $k_z^2 > 0$, $\varepsilon_1\mu_1 > 0$ case, but differs in the $k_z^2 > 0$, $\varepsilon_1\mu_1 < 0$ case. The presence of the "left-handed" and "right-handed" surface waves, together with the nontrivial behavior of the surface refractive indices (27) suggests that 1D interfaces between 2D interfaces with different properties, i.e., 1D edges between four media with $\varepsilon_{1,2,3,4}$ and $\mu_{1,2,3,4}$, could support 1D edge Maxwell modes; this is a problem for future studies.

Supplementary References

1. Bliokh, K. Y., Smirnova, D. & Nori, F. Quantum spin Hall effect of light. *Science* **348**, 1448–1450 (2015).
2. El-Ganainy, R. *et al*. Non-Hermitian physics and PT symmetry. *Nature Phys.* **14**, 11–19 (2018).
3. Heiss, W. D. The physics of exceptional points. *J. Phys. A: Math. Theor.* **45**, 444016 (2012).
4. Veselago, V. G. The electrodynamics of substances with simultaneously negative values of ε and μ. *Sov. Phys. Uspekhi* **10**, 509–514 (1968).
5. Smith, D. R., Pendry, J. B. & Wiltshire, M. C. K. Metamaterials and negative refractive index. *Science* **305**, 788–792 (2004).
6. Alù, A., Engheta, N., Erentok, A., & Ziolkowski, R. W. Single-negative, double-negative, and low-index metamaterials and their electromagnetic applications. *IEEE Antennas and Propagation Magazine* **49**, 23–36 (2007).
7. Jackson, J. D. *Classical Electrodynamics*, 3rd ed. (Wiley, New York, 1999).
8. Alpeggiani, F., Bliokh, K. Y., Nori, F. & Kuipers, L. Electromagnetic helicity in complex media. *Phys. Rev. Lett.* **120**, 243605 (2018).
9. Silveirinha, M. G. Chern invariants for continuous media. *Phys. Rev. B* **92**, 125153 (2015).
10. Brody, D. C. Biorthogonal quantum mechanics. *J. Phys. A: Math. Theor.* **47**, 035305 (2014).
11. De Nittis, G. & Lein, M. The Schrödinger formalism of electromagnetism and other classical waves – How to make quantum-wave analogies rigorous. *Ann. Phys.* **396**, 579–617 (2018).
12. Heiss, W. D. The physics of exceptional points. *J. Phys. A: Math. Theor.* **45**, 444016 (2012).
13. Dembowski, C. *et al*. Observation of a chiral state in a microwave cavity. *Phys. Rev. Lett.* **90**, 034101 (2003).
14. Peng, B. *et al*. Chiral modes and directional lasing at exceptional points. *PNAS* **113**, 6845–6850 (2016).
15. Gao, T. *et al*. Chiral modes at exceptional points in exciton-polariton quantum fluids. *Phys. Rev. Lett.* **49**, 065301 (2018).
16. Silveirinha, M. & Engheta, N. Tunneling of electromagnetic energy through subwavelength channels and bends using ε-near-zero materials. *Phys. Rev. Lett.* **97**, 157403 (2006).
17. Liu, R. *et al*. Experimental demonstration of electromagnetic tunneling through an epsilon-near-zero metamaterial at microwave frequencies. *Phys. Rev. Lett.* **100**, 023903 (2008).
18. Edwards, B., Alù, A., Young, M.E., Silveirinha, M. & Engheta, N. Experimental verification of epsilon-near-zero metamaterial coupling and energy squeezing using a microwave waveguide. *Phys. Rev. Lett.* **100**, 033903 (2008).
19. Bialynicki-Birula, I. & Bialynicki-Birula, Z. Berry's phase in the relativistic theory of spinning particles. *Phys. Rev. D* **35**, 2383–2387 (1987).
20. Berard, A. & Mohrbach, H. Spin Hall effect and Berry phase of spinning particles. *Phys. Lett. A* **352**, 190–195 (2006).
21. Bliokh, K. Y., Alonso, M. A., Ostrovskaya, E. A. & Aiello, A. Angular momenta and spin-orbit interaction of nonparaxial light in free space. *Phys. Rev. A* **82**, 063825 (2010).
22. Sheng, D. N., Weng, Z. Y. Sheng, L. & Haldane, F. D. M. Quantum spin-Hall effect and topologically invariant Chern numbers. *Phys. Rev. Lett.* **97**, 036808 (2006).
23. Hasan, M. Z. & Kane, C. L. Topological insulators. *Rev. Mod. Phys.* **82**, 3045–3067 (2010).
24. Kunst, F. K., Edvardsson, E., Budich, J. C. & Bergholtz, E. J. Biorthogonal bulk-boundary correspondence in non-Hermitian systems. *Phys. Rev. Lett.* **121**, 026808 (2018).
25. Yao, S. & Wang, Z. Edge states and topological invariants of non-Hermitian systems. *Phys. Rev. Lett.* **121**, 086803 (2018).

26. Zayats, A. V., Smolyaninov, I. I. & Maradudin, A. A. Nano-optics of surface plasmon polaritons. *Phys. Rep.* **408**, 131–314 (2005).
27. Smith, D. R., Pendry, J. B. & Wiltshire, M. C. K. Metamaterials and negative refractive index. *Science* **305**, 788–792 (2004).
28. Alù, A., Engheta, N., Erentok, A., & Ziolkowski, R. W. Single-negative, double-negative, and low-index metamaterials and their electromagnetic applications. *IEEE Antennas and Propagation Magazine* **49**, 23–36 (2007).
29. Bliokh, K. Y., Bliokh, Y. P., Freilkher, V., Savel'ev, S. & Nori, F. Unusual resonators: Plasmonics, metamaterials, and random media. *Rev. Mod. Phys.* **80**, 1201–1213 (2008).
30. Darmanyan, S. A., Neviere, M. & Zakhidov, A. A. Surface modes at the interface of conventional and left-handed media. *Opt. Commun.* **225**, 233–240 (2003).
31. Shadrivov, I.V. *et al*. Nonlinear surface waves in left-handed materials. *Phys. Rev. E* **69**, 016617 (2004).
32. Kats, A. V., Savel'ev, S., Yampol'skii, V. A. & Nori, F. Left-handed interfaces for electromagnetic surface waves. *Phys. Rev. Lett.* **98**, 073901 (2007).
